# Altered brain ion gradients following compensation for elevated CO_2_ are linked to behavioural alterations in a coral reef fish

**DOI:** 10.1038/srep33216

**Published:** 2016-09-13

**Authors:** R. M. Heuer, M. J. Welch, J. L. Rummer, P. L. Munday, M. Grosell

**Affiliations:** 1 University of Miami, RSMAS, 4600 Rickenbacker Causeway, Miami, FL 33149, USA; 2 University of North Texas, 1511 West Sycamore, Denton, TX 76203, USA; 3 Australian Research Council Centre of Excellence for Coral Reef Studies, James Cook University, Townsville, Queensland, 4811, Australia; 4 College of Marine and Environmental Sciences, James Cook University, Townsville, QLD 4811, Australia

## Abstract

Neurosensory and behavioural disruptions are some of the most consistently reported responses upon exposure to ocean acidification-relevant CO_2_ levels, especially in coral reef fishes. The underlying cause of these disruptions is thought to be altered current across the GABA_A_ receptor in neuronal cells due to changes in ion gradients (HCO_3_^−^ and/or Cl^−^) that occur in the body following compensation for elevated ambient CO_2_. Despite these widely-documented behavioural disruptions, the present study is the first to pair a behavioural assay with measurements of relevant intracellular and extracellular acid-base parameters in a coral reef fish exposed to elevated CO_2_. Spiny damselfish (*Acanthochromis polyacanthus*) exposed to 1900 μatm CO_2_ for 4 days exhibited significantly increased intracellular and extracellular HCO_3_^−^ concentrations and elevated brain pH_i_ compared to control fish, providing evidence of CO_2_ compensation. As expected, high CO_2_ exposed damselfish spent significantly more time in a chemical alarm cue (CAC) than control fish, supporting a potential link between behavioural disruption and CO_2_ compensation. Using HCO_3_^−^ measurements from the damselfish, the reversal potential for GABA_A_ (*E*_GABA_) was calculated, illustrating that biophysical properties of the brain during CO_2_ compensation could change GABA_A_ receptor function and account for the behavioural disturbances noted during exposure to elevated CO_2_.

Concerns about the impact of ocean acidification on marine ecosystems has led to a growing number of studies examining the effects of elevated CO_2_ exposure on fish^1^. While some investigated endpoints such as survival and growth appear to be relatively insensitive to projected future CO_2_ levels^2^,^3^, significant effects of elevated CO_2_ include alterations to mitochondrial function^4^,^5^, metabolic rate^6^, otolith growth^7^,^8^, reproduction^9^,^10^, and acid-base balance^11^,^12^. Perhaps the most frequently reported and consistently adverse response to elevated CO_2_ exposure in fish is disruption to sensory or cognitive function. Impairments to olfaction^13^,^14^,^15^, hearing^16^, vision^17^,^18^, lateralization^19^,^20^,^21^, and learning^22^,^23^ in fish at ocean acidification relevant CO_2_ levels demonstrate that CO_2_ broadly affects central neuronal processing. Neurosensory impacts are particularly concerning since these traits appear to show limited capacity for acclimation^13^. Furthermore, fish living near highly acidic natural CO_2_ vent systems that presumably experience high CO_2_ on a regular basis also exhibit abnormal behavioural responses^24^. Considering the rapid rate of acidification^25^ and the low CO_2_ threshold level needed to induce sensory and neurological responses (~600–800 μatm CO_2_)^26^, understanding the physiological mechanism underlying these responses is crucial for assessing risk to fish populations and could aid in predicting adaptive capacity.

Most studies to date suggest that significant effects of CO_2_, including behavioural disturbances, result from compensation that fish perform in response to a CO_2_-induced respiratory acidosis. Following exposure to elevated CO_2_, fish correct plasma and tissue pH by sustaining elevated HCO_3_^−^ levels in intracellular and extracellular fluids^12^,^27^,^28^,^29^. Although pH is corrected to pre-exposure levels, HCO_3_^−^ and PCO_2_ consequently remain elevated throughout high CO_2_ exposure. Increased plasma HCO_3_^−^ concentrations are often paired with a corresponding decline in Cl^−^ 27,30,31. Surprisingly, examination of CO_2_ acid-base balance disturbances and associated compensatory mechanisms have only been performed at ocean acidification relevant scenarios in a limited number of studies^5^,^12^,^32^,^33^.

In 2012, Nilsson and colleagues reported a series of seminal experiments on fish, suggesting compensation for elevated CO_2_ affects olfaction and lateralization by disrupting the function of the GABA_A_ receptor^34^. Under most circumstances, the GABA_A_ receptor and its associated neurotransmitter (GABA) are thought to be largely responsible for inhibitory responses throughout the vertebrate nervous system. In the model proposed by Nilsson and colleagues, following stimulation by GABA, HCO_3_^−^ and/or Cl^−^ ions enter the cell through the GABA_A_ receptor under control conditions, leading to cellular hyperpolarization, and a concomitant inhibitory response that is associated with a normal behavioural phenotype in fish. However, expected changes in extracellular and/or intracellular HCO_3_^−^ and Cl^−^ that occur during CO_2_ compensation are thought to reverse ion movement through the GABA_A_ receptor, leading to a depolarizing excitatory response and a disrupted behavioural phenotype^34^. Alleviation of olfactory and lateralization disturbances in CO_2_-exposed fish upon treatment with gabazine, a competitive GABA_A_ receptor antagonist that presumably closes the GABA_A_ receptor, implicated GABA_A_ receptor involvement in the impaired behavioural responses induced by elevated CO_2_. Since this initial study, the apparent link between CO_2_-induced behavioural disturbances and the GABA_A_ receptor has been supported by several other studies examining a variety of species (tropical and temperate, marine and freshwater), utilizing an array of sensory and behavioural assays as well as different GABA_A_ receptor antagonists and agonists^17^,^21^,^22^,^35^,^36^,^37^,^38^. Similar behavioural effects of high CO_2_ exposure that are restored by GABA_A_ receptor antagonists have also been observed in marine invertebrates^39^. Further support for the role of the GABA_A_ receptor in abnormal behaviour during CO_2_ exposure has been provided by theoretical calculations of the GABA_A_ receptor equilibrium potential (*E*_GABA_)^1^ using HCO_3_^−^ values estimated from the Gulf toadfish^12^. However, it is important to keep in mind that altered ion gradients due to high CO_2_ exposure would not necessarily have to cause a complete reversal of current to invoke a behavioural change. Even an attenuation of the normal inhibitory response of the GABA_A_ receptor due to changes in ion gradients could alter the function of neurons and account for noted behavioural disruptions.

Behavioural assays paired with GABA_A_-targeted drug treatments have strongly supported the argument that altered ion gradients in a high CO_2_ environment change the function of the GABA_A_ receptor; however, adjustments to acid-base parameters that would reverse or attenuate the current through the GABA_A_ receptor have yet to be measured in a marine fish showing a behavioural disruption. Accordingly, the aim of this study was to test the hypothesis that altered intracellular and extracellular HCO_3_^−^ due to CO_2_ compensation occurs in a species that also exhibits a behavioural disturbance when exposed to elevated CO_2_. The first objective of this study was to measure intracellular whole-brain HCO_3_^−^ and pH (pH_i_) and extracellular HCO_3_^−^ levels in blood plasma of the spiny damselfish (*Acanthochromis polyacanthus*) exposed to either control or 1900 μatm CO_2_. A second objective was to confirm that spiny damselfish exposed to the applied CO_2_ level displayed altered behavioural responses to olfactory cues as previously reported^13^,^15^. The third and final objective was to apply the measured values in an assessment of GABA_A_ receptor function by calculating *E*_GABA_ in control and CO_2_-exposed fish. To our knowledge, this is the first study to report direct measurements of both intracellular and extracellular HCO_3_^−^ and intracellular pH_i_ in a coral reef fish species.

## Results

### Physiological measurements: Brain and plasma analyses

**Figure 1:**
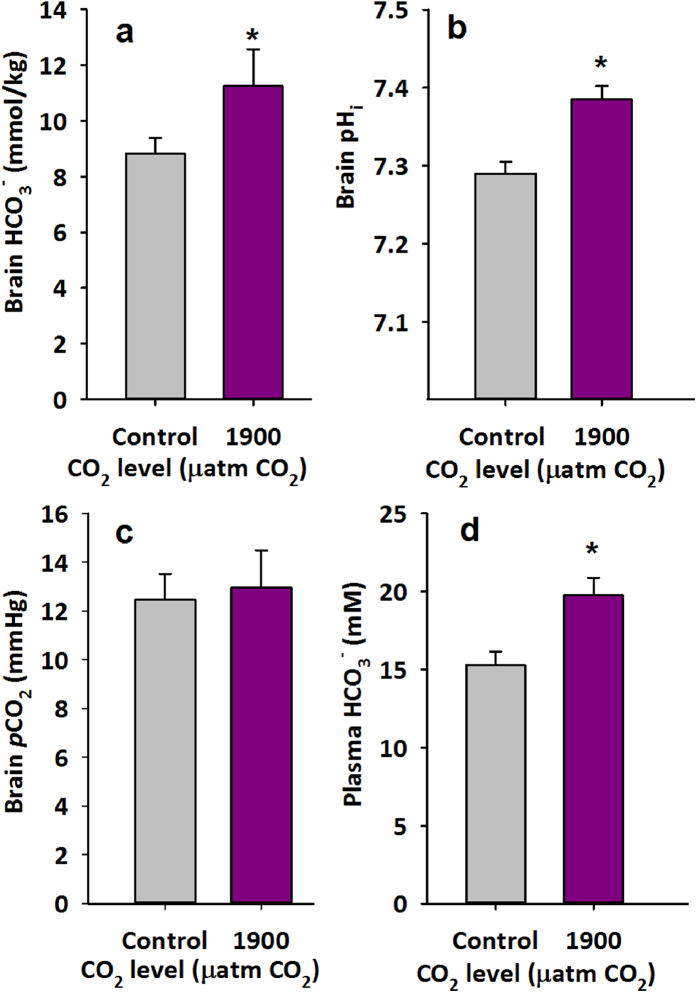
Brain and plasma analysis in the spiny damselfish exposed to high CO_2_: (**a**) Brain HCO_3_^−^ (mmol/kg tissue), (**b**) intracellular pH and C) PCO_2_ (Means ± s.e.m.; n = 8 for PCO_2_ and n = 8 and n = 6 for control and 1900 μatm CO_2_, respectively for HCO_3_^−^ and intracellular pH) in spiny damselfish (*Acanthochromis polyacanthus*) exposed to either control or 1900 μatm CO_2_ for 4 days. D) Plasma HCO_3_^−^ (mM) (Means ± s.e.m.; N = 7) of spiny damselfish (*Acanthochromis polyacanthus*) exposed to either control (value) or 1900 μatm CO_2_ for 4 days. *Denotes statistical significance from respective control value at P < 0.05. Brain HCO_3_^−^ was assessed with one-tailed t-test. N = 8 for PCO_2_ and n = 8 and n = 6 for control and 1900 μatm CO_2_, respectively for HCO_3_^−^ and intracellular pH.

As expected, brain HCO_3_^−^ (mmol/kg) and brain pH_i_ (Fig. 1a,b) were both significantly higher in damselfish exposed to 1900 μatm CO_2_ for 4 days when compared to controls (Fig. 1a,b; brain HCO_3_^−^, P < 0.03, brain pH_i_, P < 0.001). Using these values and pK’ and solubility constants from Boutilier and colleagues^40^, the brain PCO_2_ was calculated using the Henderson-Hasselbalch equation and displayed no significant difference between control and CO_2_-exposed fish (Fig. 1c). Plasma HCO_3_^−^ (mM) was also significantly higher in CO_2_-exposed fish when compared to controls (P < 0.008, Fig. 1d). Due to low blood volumes and small fish size, low plasma volume precluded measurements of pH, preventing PCO_2_ calculations for extracellular fluids. In order to verify the high levels of HCO_3_^−^ found for both brain and plasma readings a series of validation procedures were performed (Fig. S1). Measurements of a series of blanks (NaCl solution only, 50 mM) and bicarbonate standards in the range of values measured in the brain and plasma samples indicated near perfect agreement between expected and measured values. HCO_3_^−^ standards were made by diluting a 10 mmol l^−1^ NaHCO_3_^−^ solution into the NaCl solution used for determination of blank/background levels. An observed, low level of background HCO_3_^−^, represented by the constant offset from a predicted 1:1 slope, is shown in Supplementary Fig. S1. This offset was nearly negligible (60 nmol) but was nonetheless subtracted from all reported values.

### Behavioural response to a chemical alarm cue

**Figure 2:**
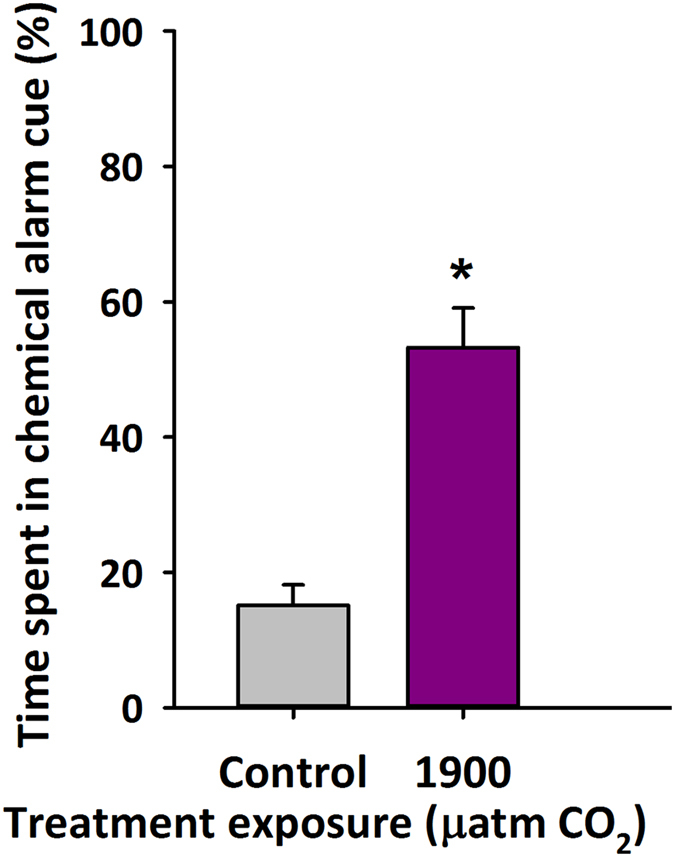
Response to alarm cues under high CO_2_ in the spiny damselfish: Percent time (mean ± s.e.m) spent in chemical alarm cue using a two-flume choice chamber for spiny damselfish (*Acanthochromis polyacanthus*) exposed to control or 1900 μatm CO_2_ for 4 days. Fish (N = 20) were offered either control water or water containing a chemical alarm cue in the choice chamber. *Denotes statistical significance from respective control value at P < 0.05.

Fish that were maintained in the laboratory at 1900 μatm CO_2_ for 4 days, when tested in a two-choice flume chamber, spent approximately half (53%) of their time in conspecific chemical alarm cue rather than untreated water. In contrast, control fish spent only ~15% in the CAC (Fig. 2, P < 0.001).

## Discussion

As expected, our results show that spiny damselfish, compensate for a CO_2_ induced acidosis by elevating plasma and brain HCO_3_^−^ following exposure to 1900 μatm CO_2_ for 4 days (Fig. 1). Also as predicted, this compensatory response appears to be associated with a reduction in chemical alarm cue avoidance behaviours (Fig. 2), suggesting impaired olfaction and/or central neuronal processing. The magnitude of HCO_3_^−^ change between control and CO_2_ exposed fish (2.4 mM) compares to that found in white muscle in both the rockcod (2.7 mM)^5^ and the toadfish (3.2 mM)^1^,^12^ at similar CO_2_ levels (1900–2000 μatm CO_2_). Control measurements of brain HCO_3_^−^ (8.8 mM) were at the high end of ranges calculated or measured for tissues in other species (0.5–9.2 mM)^5^,^41^,^42^,^43^. As evident from the equation below, the relative rather than absolute changes in ions both inside and outside the cell are relevant in assessing whether or not CO_2_ compensation alters the function of the GABA_A_ receptor. Accordingly, measurements of plasma HCO_3_^−^ were also obtained, allowing for better assessment of gradients across neuronal cell membranes. The relative difference in plasma HCO_3_^−^ concentrations between control and CO_2_ exposed fish (~4.5 mM HCO_3_^−^) was similar to that seen in toadfish (∆3.3 mM; 1900 μatm CO_2_)^12^, marbled rockcod (∆3.2 mM; 2000 μatm CO_2_)^5^, spotted catshark (∆3.0 mM; 1000 μatm CO_2_)^32^, red drum (~∆2.0 mM; 1000 μatm CO_2_)^33^, and the epaulette shark (~∆2.0 mM; 880 μatm CO_2_)^44^. However, the absolute HCO_3_^−^ levels for both control (15.3 mM) and 1900 μatm CO_2_ (19.8 mM) exposed damselfish were high compared to values reported in other species, ranging from ~3 mM up to ~11.3 mM in control animals^5^,^12^,^29^,^30^,^31^,^33^,^41^,^42^,^43^,^44^,^45^,^46^. Titration of known standards showed high accuracy. Therefore, other explanations for the high plasma HCO_3_^−^ concentrations may include the blood sampling procedure used. Ideally, blood samples for consideration of acid-base balance parameters should be taken from cannulated resting and unstressed fish under gastight conditions, an option that was unavailable in the present study due to the small size of the damselfish. Rather, blood was obtained by caudal puncture. Potential errors associated with caudal puncture can result from fish being anesthetized and briefly air-exposed during sampling, preventing CO_2_ excretion, and could lead to an overestimation of plasma HCO_3_^−^ concentrations^47^. However, a recent study using red drum (*Sciaenops oceallatus*) comparing plasma samples obtained from cannulated fish and those obtained by caudal puncture revealed the similar CO_2_-induced increase in HCO_3_^−^. Furthermore, the comparisons revealed that caudal puncture caused an increase of about 1.5 mM or ~20% HCO_3_^−^ in plasma HCO_3_^−^ compared to values obtained from catheters^33^. On balance, the levels reported here may overestimate true plasma HCO_3_^−^ levels in damselfish. Nonetheless, the increase in plasma HCO_3_^−^ observed in response to CO_2_ is a product of ambient conditions, since sampling procedures were identical for control and CO_2_-exposed fish. Brain HCO_3_^−^ measurements in the present study were not associated with the same potential errors inherent with plasma measurements but were also at the high end of reported ranges for tissue HCO_3_^−^ in other species.

Interestingly, pH_i_ in the brain of high CO_2_-exposed fish was significantly higher than in control fish (∆0.095), demonstrating a pH_i_ overshoot, a response common across many organs, species, and CO_2_ levels^5^,^12^,^48^. In the limited number of studies measuring intracellular pH at similar CO_2_ levels, white muscle and liver of the marbled rockcod show compensation with no overshoot (2000 μatm CO_2_)^5^, while the white muscle of the Gulf toadfish exhibit a pH_i_ overshoot of a similar magnitude (~∆0.07; 1900 μatm CO_2_)^12^ as seen in the damselfish brain. As suggested in Esbaugh *et al*.^12^, such an overshoot could result from active intracellular regulation to take up HCO_3_^−^ from extracellular fluids or could merely reflect passive uptake due to higher HCO_3_^−^ levels in extracellular fluids. Given the relatively high levels of plasma HCO_3_^−^ reported in this study, either explanation seems possible. Regardless of the underlying cause, it is clear that HCO_3_^−^ availability is not a limiting factor for intracellular compensation for elevated CO_2_. Further investigation into the downstream impacts of pH_i_ protection during CO_2_ exposure is needed to fully understand the tradeoffs between acid-base and neuronal homeostasis. Interestingly, the overshoot of pH_i_ in damselfish brains means that estimated PCO_2_ levels were not significantly elevated despite elevated ambient CO_2_ and elevated intracellular HCO_3_^−^ concentrations (Fig. 1).

Measurements in the present study, the first of their kind, showing altered intracellular and extracellular HCO_3_^−^ in a marine species that also exhibits a CO_2_-induced disturbance to olfactory-mediated behaviour lends support to the hypothesis that altered ion gradients can affect GABA_A_ receptor function^34^. These direct measurements also, also for the first time, provide the advantage of allowing for calculation of the reversal potential for the GABA_A_ (*E*_GABA_) under control conditions and high CO_2_ conditions. The addition of gabazine (GABA_A_ receptor antagonist) has alleviated CO_2_ induced behavioral impairments in many species to date^17^,^21^,^22^,^34^,^36^,^37^,^38^, suggesting GABA_A_ is impacted during CO_2_ exposure. Thus, results from these calculations lend support to previous reports of behavioural disturbances. One caveat to this approach, is that GABA_A_ receptor function was not directly tested in the present study, as previous studies have done primarily using the addition of gabazine. However, gabazine has been found to attenuate impairment to retinal function in this study species^17^, and has alleviated behavioral impairments in closely related damselfish species studied to date^22^,^34^.

**Figure 3:**
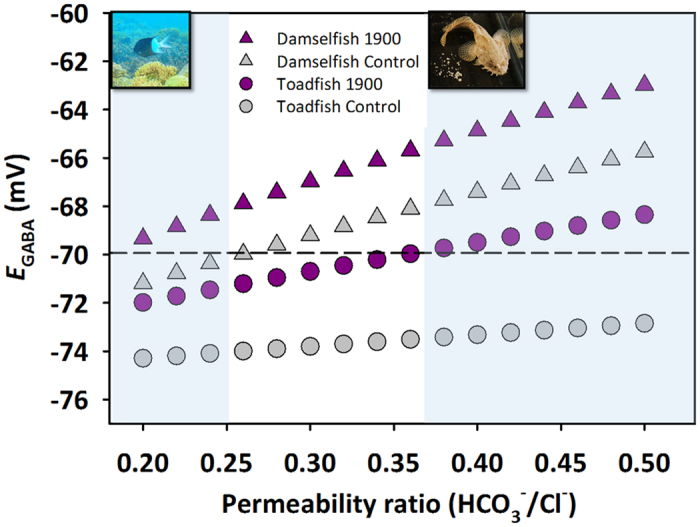
Calculated *E*_*GABA*_ values based on equation (1) over a range of physiologically relevant HCO_3_^−^/Cl^−^ permeability ratios for the GABA_A_ receptor in the spiny damselfish and the Gulf toadfish: For damselfish, brain and plasma HCO_3_^−^ concentrations from Figs 1 and 2 were used for [HCO_3_^−^]_i_ (intracellular) and [HCO_3_^−^]_o_ (extracellular), respectively. Toadfish values were taken from^12^ and modeled after calculations in^1^ using [Cl^−^]_i_ of 8 mM^50^. Permeability ratios were chosen to represent a range of values previously reported to be physiologically relevant; see review^49^. For both species, values for extracellular Cl^−^ were assumed to be 150 mM^65^, a typical value for marine teleosts and was adjusted assuming equimolar exchange of HCO_3_^−^ and Cl^−^ between extracellular fluids and the environment that has been demonstrated to occur during CO_2_ exposure in other teleosts^30^,^31^. Intracellular [Cl^−^] was assumed to be 8 mM^50^. Study temperatures of 27 °C and 25 °C were used for the calculations for damselfish and toadfish, respectively. A standard resting neuronal membrane potential of −70 mV was used to assess divergence from resting. Shaded areas represent the range of permeability ratios in which calculated *E*_GABA_ diverges in opposite directions from the resting membrane potential for the species pictured. CO_2_-exposed fish with a calculated *E*_GABA_ above the dashed line would show an abnormal depolarizing or excitatory response to GABA corresponding to an abnormal behavioural phenotype. Values used for calculations are summarized in Supplementary Table S2.

Modeled after calculations described previously (Heuer and Grosell 2014), *E*_GABA_ was calculated using the following equation^49^:


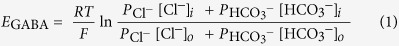


where *R* is the ideal gas constant, *T* is the absolute temperature, *F* is Faraday’s constant, and *P* represents the relative permeability of the GABA_A_ receptor for HCO_3_^−^ and Cl^−^. Intracellular and extracellular values for HCO_3_^−^ (Fig. 1.) were used to calculate *E*_GABA_ for damselfish. Extracellular Cl^−^ was assumed to be 150 mM, a typical value for marine teleosts. Under high CO_2_ conditions, HCO_3_^−^ is generally assumed to increase in extracellular fluids with a corresponding decrease in Cl^−^ 27,30,31. Thus, the increase in HCO_3_^−^ in high CO_2_ was used to adjust extracellular Cl^−^. Intracellular Cl^−^ was chosen to be 8 mM, within the range of values reported from a recent review (6–14 mM)^50^. GABA_A_ exhibits conductance for both HCO_3_^−^ and Cl^−^ in the physiological range, but tends to be more permeable to Cl^−^ 51. Different permeability ratios (*P*) have been measured in neurons in invertebrates and mammals ranging from ~0.18–0.6^49^. Since values have not been reported for fish, *E*_GABA_ was calculated over a representative range of permeability ratios (0.2–0.5) (Fig. 3). All input variables applied in the calculations presented in Fig. 3 are summarized in Supplementary Table S2.

Calculated *E*_GABA_ values for damselfish exposed to control and 1900 μatm CO_2_ conditions show a divergent deviation from the commonly assumed resting neuronal membrane potential (−70 mV) under a range of physiologically relevant permeability ratios (0.2–0.25; Fig. 3). In these instances, *E*_GABA_ for control damselfish shows a negative deviation from resting, likely conferring a normal hyperpolarizing and inhibitory response. In contrast, *E*_GABA_ for high CO_2_-exposed damselfish shows a positive deviation from resting, illustrating the potential for an abnormal depolarizing and excitatory response. In addition to damselfish, the potential for divergent responses from resting potential using calculations of *E*_GABA_ using acid-base parameters has also been estimated for the Gulf toadfish^1^. Using calculated or measured HCO_3_^−^ levels from a previous study^1^,^12^ and assuming the same [Cl^−^]_i_ level (8 mM) as the damselfish, toadfish also show a divergent deviation from the resting membrane potential, however, over a higher range of permeability ratios (0.38–0.5, Fig. 3). Finally, intracellular and extracellular values of HCO_3_^−^ have also been calculated for white muscle in a polar fish, the marbled rockcod (*Notothenia rossii*) exposed to 2000 μatm CO_2_. Using these measurements as a proxy for the brain, it appears that at 8 mM [Cl^−^]_i_, no divergent response would be noted between control and high CO_2_ exposed fish. However, if Cl^−^ is assumed to be at a lower value in the physiological range (6 mM), divergent responses are noted over a wide range of permeability ratios (0.26–0.48; Supplementary Table S2). Thus, under a given set of physiologically relevant scenarios in tropical, subtropical, and polar fish, divergent responses of currents though GABA_A_ are noted with ocean acidification relevant CO_2_ exposure levels. The above calculations and assumptions illustrate that reversal of current through the GABA_A_ receptor may occur in fish exposed to climate change relevant CO_2_ levels, which may underpin the altered behavioural responses. However, it should be noted that even shifts in the degree of a hyperpolarizing current in response to GABA_A_ rather than a full reversal could alter behaviour, as it could lead to attenuated inhibitory effects of GABA.

Although using the *E*_GABA_ model in the present study is likely an oversimplification of a complex response, it may provide a useful tool in formulating hypotheses about patterns of behavioural disturbance. The temperate wrasse^52^ and the Atlantic cod^53^ are both species that do not exhibit certain behavioural alterations following CO_2_ exposure and would be useful to examine in this context. A testable prediction is that these species do not exhibit drastic alterations of HCO_3_^−^ gradients across neuronal cell membranes during CO_2_ exposures where no behavioural alterations are observed. On the same note, *E*_GABA_ may be useful to further investigate species that show large amounts of variation in response at a particular CO_2_ level. For example, olfactory responses in the damselfish (*Pomacentrus wardii*) exposed to 700 μatm CO_2_^14^ show a large degree of variation in response among individuals. Here, individuals displaying behavioural abnormality would be predicted to have more pronounced alterations of HCO_3_^−^ gradients than those displaying normal behaviour during CO_2_ exposure. Interestingly, the percent time spent in the chemical alarm cue (53%) at 1900 μatm CO_2_ was less than observed at 1000 μatm CO_2_ in Welch *et al*.^13^ (~80%) and also in a small number of fish behaviourally tested at 1000 μatm CO_2_ during the present study (84%, n = 8, data not shown). Although not tested in this study, these findings suggest that the behavioural response to altered HCO_3_^−^ and pH_i_ may be non-linear. Since the *E*_GABA_ calculations are temperature-dependent, it also invites hypotheses under different climate change scenarios. For example, *E*_GABA_ calculated using HCO_3_^−^ measurements from the polar marbled rockcod experiencing combined temperature and CO_2_ stressors (7 °C) also showed divergence from resting membrane potential at 6 mM [Cl]_i_, but over a more narrow range of permeability ratios (0.34–0.44) than with CO_2_ alone (0.26–0.48) suggesting that elevated temperature may alleviate or reduce behavioural disturbances in this species (see values in Supplementary Table S2). Admittedly, use of the above framework would be strengthened with measured values for intracellular chloride and GABA_A_ receptor permeability in fish.

In conjunction with physiological measurements demonstrating altered ion gradients, there are several other factors that would aid in fully elucidating the mechanism underlying neurological disruption in fish. The GABA_A_ receptor can vary in subunit composition which has already been predicted to confer ion permeability differences^49^. At least in mammals, subunit composition can vary among brain regions^49^,^54^,^55^, with age^54^, and developmental stage^54^. It would be useful to know the distribution and subunit composition of GABA_A_ receptors in fish species with noted behavioural impacts. In addition, ocean acidification could also lead to regulation of neuronal transporters and enzymes involved in HCO_3_^−^ and Cl^−^ transport and is another area of fruitful research. Identification of such regulatory responses could elucidate targeted pathways for selection under future CO_2_ scenarios. Finally, it is important to note that the involvement of other receptors or neural pathways in CO_2_-induced behavioural disturbances have yet to be explored. Future work on GABA_A_ receptors and potentially other receptors known to mediate behavioural responses in the nervous system in isolated cells from CO_2_-exposed fish would aid in interpreting the mechanism underlying CO_2_ induced behavioural alterations.

In summary, this study is the first to demonstrate CO_2_ compensation using direct measurements of extracellular and intracellular HCO_3_^−^ values in a coral reef species known to exhibit a behavioural disturbance. Using these measurements, calculations of *E*_GABA_ demonstrate that an alteration of ion movement through the GABA_A_ receptor under high CO_2_ conditions is possible, and could account for the observed behavioural changes. However, more work on the GABA_A_ receptor distribution and function would greatly aid in detailing the underlying mechanisms associated with behavioural disturbances in high CO_2_ exposed fish and invertebrates. Finally, it is important to acknowledge a short acclimation period was chosen for the present study since behavioural disturbances are induced after only 4 days of exposure, providing an opportunity to examine the physiological mechanisms underlying noted behavioral responses. Identification of such mechanisms may provide insight into adaptive capacity of species. Future studies examining these endpoints across generations and over longer acclimation periods would be useful in more accurately predicting impacts to fish populations in future acidic oceans.

## Materials and Methods

### Fish collection and acclimation

Adult spiny chromis damselfish (*Acanthochromis polyacanthus*) were collected from inshore reefs at Lizard Island on the Great Barrier Reef, Australia (14°40′S, 145°28′E) in April 2015. Fish were caught by barrier netting using SCUBA, and immediately brought back to the Lizard Island Research Station where they were maintained in flow-through seawater tanks for 24 hours prior to the onset of experiments. For brain measurements, fish were 19.1 ± 2.3 and 17.0 ± 1.6 g for control and 1900 μatm CO_2_ exposures, respectively. Fish sampled for plasma were 16.1 ± 1.4 and 15.0 ± 1.3 g for control and 1900 μatm CO_2_ exposures, respectively.

Damselfish were transferred to indoor 35L tanks at either control (ambient, ~450) or 1900 μatm CO_2_ for 4 days, a time period previously demonstrated to induce olfactory behavioural abnormalities in other reef species^14^. Fish were kept on a 12:12 light:dark cycle and at a consistent temperature (~27 °C; Supplementary Table S1). Fish were held in groups of ~20 per tank prior to physiological measurements (2 replicate tanks) and 3–4 fish per tank prior to behavioural assays (6 replicate tanks), provided with PVC pipe segments for shelters, and fed daily but fasted 24 h prior to sampling. Throughout sampling, individual fish were gently netted from treatment tanks and sacrificed using 0.02 g l^−1^ MS-222 0.2 buffered with 0.3 g l^−1^ NaHCO_3_. Minimal chase periods (<20 sec) were necessary to obtain individual fish. All experiments adhered to approved animal care protocols and collecting guidelines and in accordance with the Australia Code of Practice for the Care and Use of Animals for Scientific Purposes and the Queensland Animal Care and Protection Act 2001. (General Fisheries permit 170251, Great Barrier Reef Marine Park Authority Permit G13/35909.1, James Cook University Animal Ethics Committee Regulations permits A1828 and A2089). All experimental protocols were approved by the James Cook University Animal Ethics Committee.

### Seawater manipulation

Ocean seawater was pumped into two 60 L header tanks at the Lizard Island Research Station. One tank was bubbled with air and served as the control tank, while the second was gassed with CO_2_ to achieve ~1900 μatm CO_2_. A CO_2_-stat system (Aqua Medic AT Control System) was used to dose CO_2_ in to the header tank to maintain pH levels at the set-point necessary to achieve 1900 μatm CO_2_. Seawater from these tanks was gravity fed into experimental replicate tanks at their respective CO_2_ level (control or 1900 μatm CO_2_) where temperature (C26, Comark, Norwish UK) and pH_NBS_ (pH calibrated to National Bureau of Standards) (SevenGo Pro, Mettler Toledo, Switzerland) were recorded daily. Seawater CO_2_ in treatment tanks was cross-validated using a nondispersive infrared (NDIR) sensor (GMP343, Vaisala, Helsinki, Finland) connected to a portable CO_2_ equilibration membrane submerged in the water^56^. PCO_2_ estimated by NDIR closely matched that estimated by carbonate chemistry (Supplementary Table S1). Seawater salinity was obtained daily from the Australian Institute of Marine Science ocean monitoring sensors deployed at Lizard Island. Water samples were collected for total alkalinity (TA) three times through the experimental period. TA was measured using Gran-titrations (Metrohm 888 Titrando Titrator Metrohm, AG, Switzerland), and referenced with certified material from Dr. A.G. Dickson (Scripps Institute of Oceanography, La Jolla, CA). Values of pH_NBS_, TA, salinity, and temperature were entered into CO2SYS using the constants K1 from Merhbach *et al*.^57^ refit by Dickson and Miller^58^, and Dickson for KHSO_4_^59^ to calculate PCO_2_. Averages of salinity, temperature, pH, and carbonate system parameters are reported in Supplementary Table S1.

### Physiological measurements: Brain and plasma analysis

Immediately after fish being euthanized, the brain was quickly dissected and flash frozen in a mini mortar stored in liquid nitrogen. The tissue was powdered in the mortar using a pestle stored in liquid nitrogen attached to a cordless power tool (Cryogrinder, OPS Diagnostics, New Jersey, USA). The tissue powder was then transferred to a pre-weighed cryotube, sealed, and a final weight was taken to determine tissue mass (g). Tissue homogenization and transfer to cryotubes took place in a glove box containing a CO_2_-free atmosphere^60^. A buffer containing two metabolic inhibitors, potassium fluoride (0.16 mM) and nitrilotriacetic acid (2.9 M) and adjusted to pH 7.4 with NaOH was added to the sample (250 μl/sample) as previously outlined in Pörtner 1990^60^. This mixture was briefly vortexed, centrifuged, and immediately placed on ice. This supernatant was used for measurements of both intracellular brain pH and intracellular HCO_3_^−^ (mM/kg). Contamination of extracellular fluids by this method has been deemed negligible^60^.

A custom built gas-tight chamber fitted with an electrode (PHC4000-8, Radiometer, France) and surrounded by an acrylic thermostated sleeve was used to measure pH_i_. Buffer was used to flush out the electrode chamber twice prior to processing each sample. The chamber was then flushed once with the supernatant from the homogenized tissue to clear the buffer and after which pH was recorded on a second injection of supernatant. To determine brain HCO_3_^−^ (mM/kg), an aliquot of the supernatant (corresponding to 200–1200 nmol) was added to 10 mL of 50 mM NaCl for double endpoint titrations (see below). Brain PCO_2_ was calculated using the Henderson-Hasselbalch equation (See Supplementary Information).

Due to the inherent difficulty in sampling smaller fish and the speed required to complete brain homogenization procedures, plasma and brain samples were not taken from the same individual. Blood was drawn from the caudal vein into a heparinized syringe using a 23G hypodermic needle. Blood was briefly centrifuged for 30 sec and plasma was placed on ice for subsequent analyses.

### Double endpoint titrations

Total bicarbonate and carbonate equivalents (referred to as “HCO_3_^−^” throughout) were determined in the brain supernatant and the plasma using double endpoint titrations^61^,^62^. For all samples, an aliquot was pipetted into 10 mL of a 50 mM solution of NaCl in deionized water^63^. Following a 15-minute period where samples were bubbled with CO_2_-free gas (either nitrogen or argon) to stabilize pH, initial pH was recorded (PHC 3005-8, Radiometer Analytical). Samples were titrated while being bubbled with CO_2_-free gas using 0.01 N HCl until a stable reading at or slightly below 3.8 was determined, bubbled with CO_2_-free gas for an additional 15 min, then titrated back up to the initial pH using 0.01 N NaOH. For quality control, the measured concentration of NaOH was determined to be 0.009482 N following back titrations against certified HCl standards used in this series of titrations. Additions of acid and base titrants were dispensed manually using 2 mL microburettes (GS-1200, Gilmont Instruments). Total HCO_3_^−^ equivalents in the sample were determined by subtracting the moles of NaOH from the moles of HCl required to bring the sample back to the initial pH^63^.

### Behavioural response to a chemical alarm cue

As outlined in a previous study^13^, the response to olfactory cues was tested by giving a fish the choice between untreated seawater and seawater containing CAC in a two-channel choice flume. As the fish used in this study were considerably larger (15.8 ± 0.5 g) than those tested by Welch *et al*.^13^ (0.12 ± 0.07 g), a larger flume was used (30 cm × 13 cm)^64^. Control or CAC treated water was gravity fed into either side at a constant flow rate of 450 mL/min. Validation of equal (laminar) flow rates was achieved using both a flow meter and a dye test following each water change performed every second fish. Dye tests confirmed water streams coming from each side of the flume were not mixed (see Supplementary Fig. S2).

CAC-treated seawater was made by euthanizing a donor fish of the same species with a quick blow to the head and making shallow cuts along the side of the body to mimic an injured conspecific. The fish was then rinsed with 30 mL of seawater that was collected and added to 10 L of seawater that would serve as CAC seawater in the choice system. One donor fish was used per test fish in an experimental run. Previous work has demonstrated that the behavioural response to a chemical cue is the same when presented in control and elevated CO_2_ water^14^. This finding was confirmed in a recent study showing no effect of using control vs. treatment water flume choice and escape response behavioural assays. In addition, the aforementioned study used two species previous used in CO_2_ behavioral experiments on Lizard Island (*Amphiprion percula* and *Pomacentrus amboinensis*)^38^. Fish in the choice system in this particular study were tested using control seawater.

During choice tests, fish were introduced to the center of the downstream end of the flow chamber and allowed to acclimate for 2 minutes. Following acclimation, the location of the fish was recorded every five seconds over a 2 minute recording period. During a three-minute “rest” period, the water sources were switched to eliminate any side biases and the two-minute acclimation period was repeated. The fish was gently re-centered in the flume during this time a piece of soft mesh, and the recording period was then repeated. To avoid confounding issues of handling stress, fish tested in flume trials were not used for physiological measurements outlined above.

### Statistical analysis

Student t-tests were used to compare measurements from control and CO_2_-treated fish. Data with *a priori* directional predictions were assessed using a one-tailed t-test and are specifically noted in the text and figures. Data that were non-parametric were analyzed using a Mann-Whitney rank sum test. Significance was determined at P < 0.05 for all tests and all values are presented as means ± s.e.m.

## Additional Information

**How to cite this article**: Heuer, R. M. *et al*. Altered brain ion gradients following compensation for elevated CO_2_ are linked to behavioural alterations in a coral reef fish. *Sci. Rep.* **6**, 33216; doi: 10.1038/srep33216 (2016).

## Supplementary Material

Supplementary Information
