# Supplementary material for: Altered brain ion gradients following compensation for elevated CO2 are linked to behavioural alterations in a coral reef fish
Source: Sci Rep. 2016 Sep 13;6:33216. doi: 10.1038/srep33216 (PMC5020430; doi:10.1038/srep33216)
Supplement: Supplementary Information [file srep33216-s1.pdf]

Title: Altered brain ion gradients following compensation for elevated CO_2 are linked to behavioural alterations in a coral reef fish

Supplementary information

*Heuer, R.M.[1,2], Welch, M.J.[3,4], Rummer, J.L[3], Munday, P.L.[3], Grosell, M.[2]
[1] University of North Texas, 1511 West Sycamore, Denton, TX 76203, [2] University of Miami, RSMAS, 4600 Rickenbacker Causeway, Miami, FL 33149. [3] Australian Research Council Centre of Excellence for Coral Reef Studies, James Cook University, Townsville, Queensland, 4811, Australia. [4] College of Marine and Environmental Sciences, James Cook University, Townsville, QLD 4811, Australia
*corresponding author, rheuer@rsmas.miami.edu, 305-421-4665

Supplementary Methods

The Henderson Hasselbach equation was used to estimate pCO_2 in brain tissue from measured pH and HCO_3^- equivalents (≈total CO_2):

$$pCO_2 = [\text{total } CO_2] / (\alpha CO_2 * (10^{pH-pKa}) + \alpha CO_2)$$

For our calculations the following constants were applied: α=0.038, pKa=6.04.

Supplementary Figure S1: Verification of double endpoint titration methodology using known HCO_3^- standards: Double endpoint titrations performed on titration solution (50 mM NaCl) with no addition of HCO_3^- (0), 0.5 μmol HCO_3^-, and 1.0 μmol HCO_3^-. The chosen HCO_3^- standards bracketed the values measured from the analyzed samples. The dashed line represents perfect agreement between added and measured values. The solid line represents the linear regression performed on actual measured standards (slope=0.98, r^2=0.9991). The difference between to the two lines represents background that was corrected for in reported values.

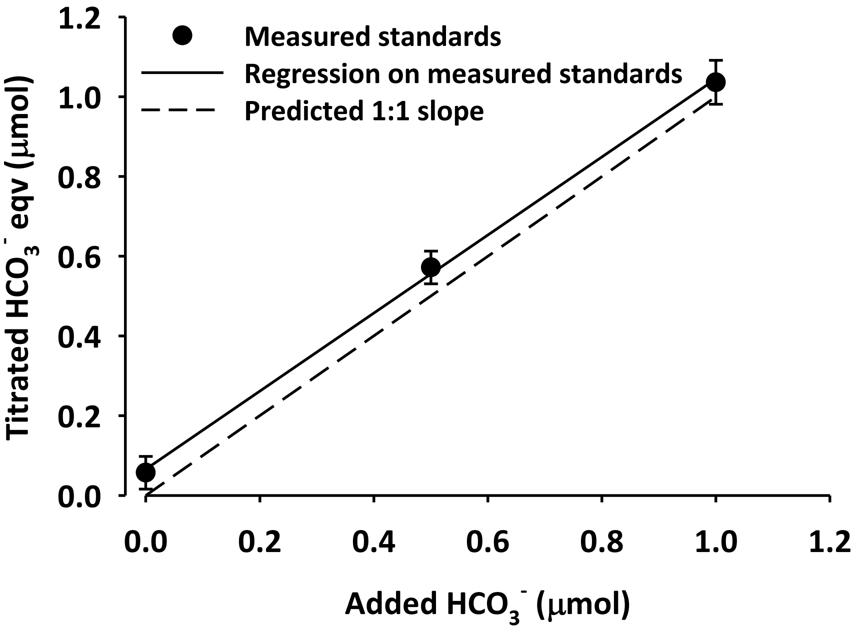

Supplementary Table S1: Water chemistry parameters. Values are presented as means ± standard deviation. PCO_2 was estimated using values of pH_{NBS}, TA, salinity, and temperature in CO2SYS using the constants K1 from Merhbach et al (1973) refit by Dickson and Miller (1987), and Dickson for $KHSO_4$ (Pierrot et al. 2006).

	pH_{NBS}	PCO_2 (μatm)	Alkalinity (μmol kg^{-1})	Salinity (p.p.t.)	Temp. (°C)	PCO_2 (NDIR) (μatm)
Control (ambient)	8.15 ± .02	431 ± 20	2277 ± 9	35.1 ± 0.05	27.4 ± 0.5	437 ± 23
1900 μatm (CO_2)	7.58 ± .02	1945 ± 96	2271 ± 8	35.0 ± 0.06	27.6 ± 0.5	1912 ± 67

Supplementary Table S2: Values used to calculate E_{GABA} in Figure 3 and to calculate E_{GABA} in a polar species under two temperature scenarios

	Temp. (°C)	$[HCO_3^-]_o$	$[HCO_3^-]_i$	$[Cl^-]_o$	$[Cl^-]_i$
Damselfish					
Control	27	15.3	8.8	150	8
1900 μatm CO_2	27	19.8	11.2	145.6	8
Toadfish					
Control	25	3.3	1.8	150	8
1900 μatm CO_2	25	6.3	5.0	147	8
Rockcod					
Control	1	8.05	3.99	150	6
2000 μatm CO_2	1	11.28	6.72	146.8	6
Rockcod					
Control	7	6.31	5.29	150	6
2000 μatm CO_2	7	10.08	6.85	146.2	6

Values used for E_{GABA} calculations (equation 1) in Figure 3. Values for toadfish were taken from (Esbaugh et al. 2012) and intracellular HCO_3^- values were calculated in (Heuer and Grosell 2014) from this data. Values for the marbled rockcod were taken from (Strobel et al. 2012) and are not presented in Figure 3.

Supplementary Figure S2: Representative dye tests using two choice flume chamber. Image shows a typical dye test using two-choice flume chamber that is representative of dye tests conducted in the present study. The test indicates that flows presented the fish with a distinct choice between two separate flows. Image credit: Michael Jarrold

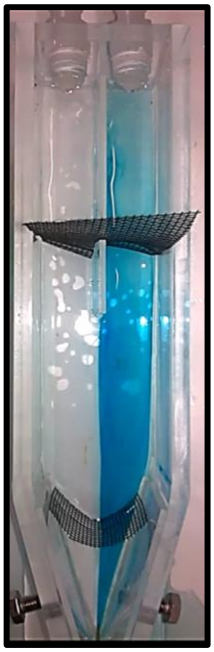

References

Esbaugh, A.J., R. Heuer and M. Grosell. 2012. Impacts of ocean acidification on respiratory gas exchange and acid-base balance in a marine teleost, Opsanus beta. Journal of Comparative Physiology B: Biochemical, Systemic, and Environmental Physiology 182: 921-934.

Heuer, R.M., and M. Grosell. 2014. Physiological impacts of elevated carbon dioxide and ocean acidification on fish. American Journal of Physiology Regulatory Integrative and Comparative Physiology 307: R1061-R1084.

Pierrot, D., E. Lewis and D. Wallace. 2006. MS Excel program developed for CO2 system calculations. ORNL/CDIAC-105a. Carbon Dioxide Information Analysis Center, Oak Ridge National Laboratory, US Department of Energy, Oak Ridge, Tennessee.

Strobel, A., S. Bennecke, E. Leo, K. Mintenbeck, H.O. Poertner and F.C. Mark. 2012. Metabolic shifts in the Antarctic fish Notothenia rossii in response to rising temperature and PCO2. Frontiers in Zoology 9.
